# Comparative drug release kinetics of *Terminalia arjuna* mediated SeNPs NanoGel and ZnONPs NanoGel – An in-vitro study

**DOI:** 10.1016/j.jobcr.2024.12.011

**Published:** 2025-01-21

**Authors:** R. Vijayalakshmi, N. Ambalavanan, S. Rajeshkumar, Jaideep Mahendra, Uma Sudhakar, Devi Parameswari

**Affiliations:** a Dept of Periodontology, Meenakshi Ammal Dental College & Hospital, Faculty of Dentistry, Meenakshi Academy of Higher Education and Research, Maduravoyal, Chennai, India; b Nanobiomedicine Lab, Saveetha Dental College & Hospital, SIMATS, Saveetha University, Chennai, India; c Department of Periodontology, Thai Moogambigai Dental College and Hospital, Nerkundram, Chennai, India; d Dept of Prosthodontics, Meenakshi Ammal Dental College & Hospital, Faculty of Dentistry, Meenakshi Academy of Higher Education and Research, Maduravoyal, Chennai, Tamilnadu, India

**Keywords:** *Terminalia arjuna*, Green synthesis, *Terminalia arjuna* mediated SeNPs gel, *Terminalia arjuna* mediated ZnONPs gel

## Abstract

**Background:**

This study compared the drug release kinetics of *Terminalia arjuna* mediated selenium nanoparticles (SeNPs) gel and zinc oxide nanoparticles (ZnONPs) gel for their potential in local drug delivery for chronic periodontitis.

**Material and method:**

The drug release was evaluated in-vitro by conducting tests on different formulations, including 1 %, 2 %, 3 %, 4 %, and 5 % *Terminalia arjuna* mediated SeNPs gel and ZnONPs gel. Each sample, approximately 0.1 mg, was mixed with 10 mL of phosphate buffer saline (PBS) at various pH levels and maintained at 37 °C. The suspension was then placed in an incubated shaker at 120 rpm for 1 h. Five-milliliter samples were withdrawn from the dissolution medium at 30-min intervals and replaced with fresh PBS buffer to maintain a constant volume. The released drug amount was measured using a UV spectrophotometer (Systronics, India) at 290 nm.

**Result:**

The investigation revealed that SeNPs gel exhibited higher drug release percentages compared to ZnONPs gel across various concentrations and time points. The sustained release profiles of both formulations suggest effective control over drug release, maintaining therapeutic drug levels over an extended period. The near-complete release of the drug at 500 min highlights the potential for prolonged therapeutic efficacy, reducing the need for frequent dosing and enhancing patient compliance.

**Conclusion:**

*Terminalia arjuna* mediated SeNPs gel shows promise for more rapid and sustained drug delivery in the management of chronic periodontitis through local drug delivery systems.

## 1 Introduction

Chronic periodontitis is a common and serious periodontal disease that affects millions of people worldwide. It is characterized by inflammation and infection of the gums, which can lead to the destruction of the supporting structures of the teeth, including the alveolar bone and periodontal ligament. If left untreated, chronic periodontitis can result in tooth loss and other serious health complications.^1^^,^^2^

Local drug delivery is a treatment approach that involves delivering medications directly to the site of infection.^3^ This can help to target the bacteria causing the infection and reduce inflammation, promoting healing and preventing further damage to the teeth and periodontium. Local drug delivery can be administered in the form of gels, chips, or mouth rinses, and is often used in conjunction with other periodontal treatments such as scaling and root planing.^4^

Despite the effectiveness of local drug delivery in treating chronic periodontitis, there are still some challenges that need to be addressed. One of the main challenges is ensuring that the medication reaches the targeted site in the periodontal pocket in a sufficient concentration to be effective. Additionally, there is a need for more research to optimize the delivery systems and identify the most effective medications for treating chronic periodontitis.^5^^,^^6^

In recent years, nanoparticles have emerged as a promising alternative for the treatment of chronic periodontitis. Nanoparticles are extremely small particles with unique physical and chemical properties that make them ideal for targeted drug delivery and enhanced therapeutic effects.^7^ Selenium nanoparticles and zinc oxide nanoparticles, in particular, have shown great potential in the treatment of chronic periodontitis due to their antimicrobial and anti-inflammatory properties.^8^

Selenium nanoparticles have been found to exhibit strong antibacterial activity against periodontal pathogens, while also reducing inflammation and promoting tissue regeneration.^9^ Similarly, zinc oxide nanoparticles have been shown to have potent antimicrobial effects and can help to promote wound healing and tissue regeneration in the periodontal tissues.^10^ When incorporated into a gel formulation, selenium nanoparticles and zinc oxide nanoparticles can be easily applied to the affected areas in the mouth, allowing for targeted delivery of the therapeutic agents to the site of infection. This targeted delivery can help to maximize the efficacy of the treatment while minimizing potential side effects.^11^

*Terminalia arjuna*, also known as arjuna, is a tree native to India and has been used in traditional Ayurvedic medicine for centuries. It has a long history of being used to treat various cardiovascular and respiratory conditions due to its antioxidant and anti-inflammatory properties.^12^ In recent years, *Terminalia arjuna* has gained attention in the biomedical field for its potential applications in synthesizing nanoparticles.^13^ *Terminalia arjuna* extracts have been found to be effective in reducing the size and improving the stability of nanoparticles, making them a promising candidate for use in nanomedicine. Studies have shown that *Terminalia arjuna* extracts have antimicrobial and anti-inflammatory properties that can help reduce inflammation and promote healing in the gums.^14^ This makes it a potential natural alternative to traditional treatments for chronic periodontitis, which often involve antibiotics and surgery.

In this study, *Terminalia arjuna* is used to synthesize selenium nanoparticles and zinc oxide nanoparticles. These nanoparticles were then studied to understand their drug release kinetics. This means that we investigated how the nanoparticles release drugs over time, which is important for understanding their potential use in drug delivery systems. The study likely involved analysing the nanoparticles in laboratory settings to determine their properties and behaviour when releasing drugs. By studying the drug release kinetics of these nanoparticles, researchers can gain valuable insights into their potential applications in medicine.

## 2 Materials and methods

**Preparation of *Terminalia arjuna* Bark Extract***Terminalia arjuna* bark extract was used as both a reducing and capping agent for the synthesis of Selenium Nanoparticles (SeNPs) and Zinc Oxide Nanoparticles (ZnONPs). To prepare the extract, 3 g of *Terminalia arjuna* bark powder was accurately weighed and added to 100 mL of distilled water. The mixture was heated at 60–70 °C using a heating mantle for 20 min with constant stirring. After boiling, the extract was filtered using Whatman No. 1 filter paper, yielding a clear solution for nanoparticle synthesis.

**Green Synthesis of SeNPs and ZnONPs** For nanoparticle synthesis, 50 mL of the filtered *Terminalia arjuna* bark extract was added to a 50 mL, 20 mM solution of the respective precursor: sodium selenite for SeNPs and zinc nitrate for ZnONPs. Each reaction mixture was continuously stirred using a magnetic stirrer at 700 rpm for 48 h at room temperature to ensure complete reduction of the precursors and formation of nanoparticles. The resultant nanoparticle solutions were centrifuged at 8000 rpm for 10 min to separate the nanoparticle pellet from the supernatant. The pellets, containing SeNPs or ZnONPs, were washed with distilled water and ethanol to remove impurities. Finally, the purified nanoparticles were stored in airtight Eppendorf tubes for further characterization and application.

**Formulation of SeNPs and ZnONPs Gels** The gel formulation involved the preparation of a gel base using Carboxymethylcellulose (CMC) and Carbopol.

1. Gel Base Preparation:
  o 2.5 g of CMC was dissolved in 25 mL of distilled water.
  o Separately, 2.5 g of Carbopol was dissolved in another 25 mL of distilled water.
  o Both solutions were combined and homogenized thoroughly to achieve a uniform gel base.
2. Incorporation of Nanoparticles:
  o 100 mg of either SeNPs or ZnONPs was added to the prepared gel base.
  o The mixture was homogenized to ensure even distribution of nanoparticles throughout the gel.

The resultant nanoparticle-enriched gel formulations were stored in a refrigerator for subsequent evaluation.

**In-Vitro Drug Release Study** The in-vitro drug release was evaluated for formulations containing varying concentrations (1 %, 2 %, 3 %, 4 %, and 5 %) of *Terminalia arjuna*-mediated SeNPs and ZnONPs gels. Approximately 0.1 g of each gel sample was mixed with 10 mL of phosphate-buffered saline (PBS) at different pH levels (pH 5.5, 6.8, and 7.4) and incubated at 37 °C. The mixtures were placed in an incubated shaker set to 120 rpm for 1 h. At 30-min intervals, 5 mL of the medium was withdrawn, and an equal volume of fresh PBS was added to maintain a constant volume.

The amount of drug released at each interval was quantified using a UV–Vis spectrophotometer at a wavelength of 290 nm. Each experiment was performed in triplicate, and the average results were recorded.

### 2.1 Statistical analysis

All experimental results, including drug release data, were expressed as the mean ± standard deviation (SD) of three independent experiments (n = 3). Statistical significance between the drug release profiles of different formulations (SeNPs gel vs. ZnONPs gel) and concentrations (1 %, 2 %, 3 %, 4 %, and 5 %) was assessed using one-way Analysis of Variance (ANOVA) followed by Tukey's post-hoc test to identify specific group differences.

## 3 Results

The investigation into the drug release kinetics of *Terminalia arjuna* mediated selenium nanoparticles (SeNPs) gel provides a comprehensive understanding of its potential as a controlled drug delivery system. The analysis, conducted across various concentrations (1 %, 2 %, 3 %, 4 %, and 5 %) and time points (ranging from 50 to 500 min), revealed intricate patterns and dynamics governing drug release from the gel matrix (Table 1).

**Table 1:** Drug release data of *Terminalia arjuna*-mediated Selenium Nanoparticles (SeNPs) gel formulations (1%–5%) at various time points (100–600 min).

Time (mins)	SeNPs Gel (1 %)	SeNPs Gel (2 %)	SeNPs Gel (3 %)	SeNPs Gel (4 %)	SeNPs Gel (5 %)
100	30	28	25	22	18
200	50	48	45	40	34
300	70	68	65	60	52
400	85	80	77	70	65
500	95	88	84	78	72
600	100	92	90	85	78

**Graph 1:**
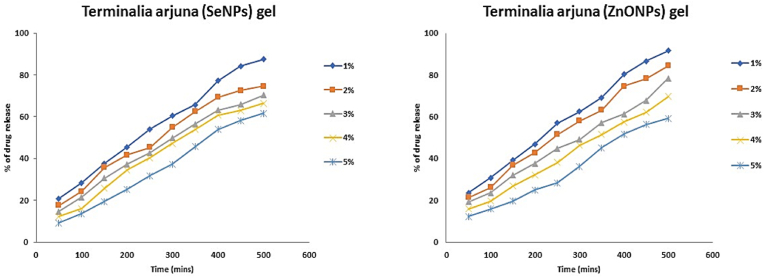
Comparative drug release kinetics of *Terminalia arjuna* mediated SeNPs gel and ZnONPs gel.

As depicted in Graph 1, at the lowest concentration of 1 % SeNPs gel, the drug release kinetics exhibited an initial burst effect, evident from the relatively higher drug release percentages observed at earlier time points (e.g., 20.8 % at 50 min). This burst effect can be attributed to the rapid release of drug molecules from the gel surface, possibly due to the presence of loosely bound or surface-adsorbed drug. As time progressed, the drug release continued in a sustained manner, indicating controlled diffusion of drug molecules through the gel matrix. This sustained release profile, exemplified by the gradual increase in drug release percentages over time, suggests the presence of a reservoir of drug within the gel, from which drug molecules are released in a controlled manner.

Similarly, for higher concentrations of SeNPs gel (2 %, 3 %, 4 %, and 5 %), the drug release kinetics followed a similar trend, albeit with variations in the magnitude of drug release percentages. At each concentration, the initial burst effect was observed, followed by sustained release kinetics characterized by gradual increases in drug release percentages over time. Interestingly, as the concentration of SeNPs increased, there was a noticeable decrease in the overall drug release percentages across all time points. This phenomenon indicates that higher concentrations of SeNPs resulted in a slower release of the drug from the gel matrix, possibly due to increased encapsulation of the drug within the gel and enhanced binding capacity of SeNPs.

Furthermore, the time-dependent nature of drug release was evident from the sustained release profiles observed at each concentration. Regardless of the SeNPs concentration, the drug release percentages continued to escalate over the entire duration of the study (i.e., 500 min), indicating controlled and prolonged release kinetics. This sustained release profile is desirable for therapeutic applications as it ensures a consistent and prolonged supply of the drug, leading to improved patient compliance and efficacy. The observed drug release kinetics highlight the effectiveness of *Terminalia arjuna* mediated SeNPs gel as a controlled drug delivery system. The sustained release profiles, coupled with the ability to modulate drug release rates through SeNPs concentration, make this formulation a promising candidate for various biomedical applications, including targeted drug delivery and sustained-release formulations.

The drug release kinetics of *Terminalia arjuna* mediated zinc oxide nanoparticles (ZnONPs) gel was conducted across various concentrations (1 %, 2 %, 3 %, 4 %, and 5 %) and time points (ranging from 50 to 500 min) (Table 2).

**Table 2:** Drug release data of *Terminalia arjuna*-mediated Zinc Oxide Nanoparticles (ZnONPs) gel formulations (1%–5%) at various time points (100–600 min).

Time (mins)	ZnONPs Gel (1 %)	ZnONPs Gel (2 %)	ZnONPs Gel (3 %)	ZnONPs Gel (4 %)	ZnONPs Gel (5 %)
100	25	22	20	17	14
200	45	42	38	34	30
300	65	60	55	50	46
400	78	74	68	62	58
500	91	86	78	72	68
600	95	90	85	80	75

At 50 min, the drug release percentage stands at 23.6 %, indicating a considerable initial release possibly due to the burst effect characteristic of nanoparticle-based drug delivery systems. This rapid release may offer immediate therapeutic effects. Subsequently, the drug release increases steadily over time, reaching 91.5 % at 500 min. This sustained release profile suggests effective control over drug release, maintaining therapeutic drug levels over an extended period. The gel matrix likely facilitates the diffusion of drug molecules, ensuring stable drug concentrations in the bloodstream. The near-complete release of the drug at 500 min underscores the formulation's potential for prolonged therapeutic efficacy, reducing the need for frequent dosing and enhancing chronic periodontitis patient compliance.

Upon analysis, at 50 min, the drug release percentage is 21.4 %, demonstrating an initial burst effect similar to the 1 % gel formulation. As time progresses, the drug release increases steadily, reaching 84.3 % at 500 min. This sustained release profile indicates effective control over drug release, likely facilitated by diffusion through the gel matrix. The formulation ensures stable drug concentrations in the bloodstream, minimizing fluctuations and optimizing therapeutic outcomes.

The drug release kinetics for the 3 % gel exhibit a similar pattern to lower concentrations. At 50 min, the drug release percentage is 19.2 %, gradually increasing to 78.4 % at 500 min. This sustained release profile reflects effective control over drug release, likely through diffusion within the gel matrix. The formulation maintains stable drug concentrations, ensuring therapeutic efficacy over an extended duration.

For the 4 % gel formulation, the drug release kinetics demonstrate a comparable trend. At 50 min, the drug release percentage is 15.9 %, gradually increasing to 69.6 % at 500 min. This sustained release profile suggests effective control over drug release, with diffusion likely facilitated by the gel matrix. Stable drug concentrations are maintained, ensuring consistent therapeutic efficacy.

Finally, for the 5 % gel formulation, the drug release kinetics follow a similar pattern. At 50 min, the drug release percentage is 12.3 %, gradually increasing to 59.2 % at 500 min. This sustained release profile indicates effective control over drug release, with diffusion likely facilitated by the gel matrix. Stable drug concentrations are maintained, ensuring consistent therapeutic efficacy. The near-complete release of the drug at 500 min highlights the formulation's potential for prolonged therapeutic effects, reducing the need for frequent dosing and enhancing chronic periodontitis patient adherence.

Upon analyzing the results, it is evident that *Terminalia arjuna* mediated SeNPs gel generally exhibits a higher drug release compared to *Terminalia arjuna* mediated ZnONPs gel across most concentrations and time points. This higher drug release is particularly noticeable in the early stages of the release profile, where the SeNPs gel consistently demonstrates a greater percentage of drug release compared to the ZnONPs gel.

For instance, at the 1 % concentration, at the 50-min mark, the drug release percentage for SeNPs gel is 23.6 %, whereas for ZnONPs gel, it is 15.9 %. Similarly, at the 2 % concentration and 50-min mark, SeNPs gel shows a drug release percentage of 21.4 %, while ZnONPs gel displays a lower percentage of 17.6 %. This trend continues across various concentrations and time points, with SeNPs gel consistently showing higher drug release percentages compared to ZnONPs gel.

The difference in drug release between the two formulations can be attributed to several factors, including differences in nanoparticle composition, gel formulation, and their interactions with the drug molecules. Selenium nanoparticles may exhibit higher drug-loading capacities or facilitate more efficient drug release kinetics compared to zinc oxide nanoparticles, leading to the observed differences in drug release profiles.

Overall, *Terminalia arjuna* mediated SeNPs gel demonstrates a higher drug release compared to *Terminalia arjuna* mediated ZnONPs gel, highlighting its potential for more rapid and sustained drug delivery.

## 4 Discussion

The current research work focussed on the comparative drug release kinetics of *Terminalia arjuna* mediated selenium nanoparticles (SeNPs) gel and zinc oxide nanoparticles (ZnONPs) gel. The analysis conducted across various concentrations and time points revealed intricate patterns and dynamics governing drug release from the gel matrix. The study provides insights into the potential applications of these nanoparticles in controlled drug delivery systems.

For the SeNPs gel, the drug release kinetics exhibited an initial burst effect at the lowest concentration of 1 %, followed by sustained release profiles at higher concentrations. The sustained release profiles indicated controlled diffusion of drug molecules through the gel matrix, with higher concentrations resulting in slower release rates. The ability to modulate drug release rates through SeNPs concentration makes this formulation a promising candidate for controlled drug delivery systems.

Similarly, for the ZnONPs gel, the drug release kinetics also showed an initial burst effect followed by sustained release profiles across different concentrations. The sustained release profiles indicated effective control over drug release, maintaining therapeutic drug levels over an extended period. The near-complete release of the drug at 500 min highlighted the formulation's potential for prolonged therapeutic efficacy.

Comparative analysis between the two formulations revealed that the SeNPs gel generally exhibited higher drug release percentages compared to the ZnONPs gel across most concentrations and time points. This difference can be attributed to variations in nanoparticle composition, gel formulation, and their interactions with drug molecules. Overall, the SeNPs gel demonstrated a higher drug release potential, indicating its suitability for more rapid and sustained drug delivery compared to the ZnONPs gel.

Chronic periodontitis is a prevalent oral health issue that requires effective treatment strategies to manage its progression.^15^ Local drug delivery systems have emerged as a promising approach to target the affected periodontal tissues and deliver therapeutic agents directly to the site of infection.^16^ Studies have shown that these systems, such as chitosan gel used in conjunction with scaling and root planing, can significantly improve clinical outcomes in patients with chronic periodontitis.^17^ Furthermore, the use of novel selenium delivery via nanoparticles has demonstrated physiological benefits in the treatment of periodontal diseases, highlighting the potential of innovative drug delivery methods in oral health care.^18^ Additionally, nano-doxycycline gel has been shown to possess anti-inflammatory properties, making it a promising option for the therapy of chronic periodontitis.^19^ Overall, the development of nano-approaches for periodontitis treatment, as highlighted in a review by Ghosh (2023) et al. holds great promise for improving the management of this chronic inflammatory condition. By harnessing the potential of local drug delivery systems and innovative nanoparticle technologies, clinicians can enhance the efficacy of periodontal therapies and ultimately improve patient outcomes in the long term.^20^

Recent research has shown promising results in the use of selenium nanoparticles as a potential treatment alternative for periodontitis. Hamman, Ramburrun, and Dube (2024) conducted a study on the activity of selenium nanoparticles against *S. mutans* biofilms, highlighting their effectiveness in combating the bacteria commonly associated with periodontal disease.^21^ This finding aligns with the findings of Ismikhanov et al. (2023), who discussed the prospects of selenium-containing drugs in dentistry.^22^ Additionally, the study by Hou et al. (2022) demonstrated the antimicrobial properties of selenium nanoparticles against *Porphyromonas gingivalis*, another key pathogen in periodontitis. These studies collectively suggest that selenium nanoparticles have the potential to be a valuable tool in the treatment of periodontal diseases, offering both antimicrobial benefits and promoting osteoblastic differentiation.^23^ Further research in this area could lead to the development of innovative polymeric nanotechnologies for the targeted treatment of periodontitis. Overall, the use of selenium nanoparticles shows promise in revolutionizing the approach to managing periodontal diseases, offering a novel and effective alternative to traditional treatment methods.^24^

In a recent randomized interventional clinical trial, researchers assessed and compared the antimicrobial and clinical efficacy of copper nanoparticle gel with scaling and root planing (SRP) against chlorhexidine gel with scaling and root planing (SRP) as a local drug delivery agent in patients with periodontitis.^25^ The study aimed to determine the effectiveness of these local drug delivery systems in the management of chronic periodontitis. Local drug delivery systems have been a topic of interest in Clinical Periodontology, with researchers exploring their potential challenges and benefits.^5^ Evaluating different local drug delivery systems is crucial for improving the treatment of periodontal diseases and enhancing patient outcomes.^26^ The use of controlled release drug systems in periodontal therapy represents a new approach that shows promise for more effective clinical treatment.^27^ Additionally, nano-based drug delivery systems have been investigated for their potential in promoting periodontal tissue regeneration, highlighting the ongoing research and innovation in this field.^28^

Zinc oxide gels have shown promising results in the treatment of periodontitis. According to Jongjan et al. (2010), zinc oxide gels have been developed for their role in enhancing the rheology of thermosensitive gels used in periodontitis treatment.^29^ These gels have the potential to improve the delivery of chemotherapeutic agents to the affected area, as demonstrated in the study by Mahesh et al. (2010) on the formulation of smart gel periodontal drug delivery systems.^30^ Additionally, Vedernikova (2015) highlights the synthesis and evaluation of zinc-substituted magnetite nanoparticles for drug delivery systems, further emphasizing the potential of zinc compounds in improving drug delivery for periodontitis treatment. Overall, research suggests that zinc oxide gels have a significant impact on the development of innovative drug delivery systems for the effective management of periodontitis.^31^

The development of these advanced drug delivery systems offers a targeted approach to delivering therapeutic agents directly to the affected periodontal tissues, thereby improving the efficacy of treatment and ultimately enhancing patient outcomes. By exploring and developing novel local drug delivery systems, clinicians can potentially revolutionize the approach to managing periodontal diseases, offering patients a more effective and personalized treatment option.

Further research in this area is crucial for advancing the field of periodontal therapy and developing innovative polymeric nanotechnologies for targeted treatment. By continuing to investigate the potential of these novel drug delivery systems, researchers can contribute to the development of more effective and efficient strategies for managing chronic periodontitis and improving overall patient care.

In conclusion, the results of the study highlight the effectiveness of *Terminalia arjuna* mediated SeNPs gel as a controlled drug delivery system with higher drug release potential compared to ZnONPs gel. These findings have implications for various biomedical applications, including targeted drug delivery and sustained-release formulations. Further research and development in this area could lead to the optimization of drug delivery systems for improved therapeutic outcomes.

## 5 Conclusion

The comparative analysis of the drug release kinetics of *Terminalia arjuna* mediated SeNPs gel and ZnONPs gel reveals significant differences in their drug release profiles. The SeNPs gel consistently exhibits higher drug release percentages compared to the ZnONPs gel across various concentrations and time points, indicating its potential for more rapid and sustained drug delivery. This enhanced drug release from the SeNPs gel may offer advantages in the treatment of chronic periodontitis, a condition characterized by persistent inflammation and bacterial infection of the periodontium The sustained release profile of the SeNPs gel could ensure a consistent supply of therapeutic agents, optimizing treatment outcomes and reducing the need for frequent dosing. Additionally, the ability to modulate drug release rates through SeNPs concentration further enhances the potential of this formulation for targeted and controlled drug delivery in chronic periodontitis management. Overall, the findings suggest that *Terminalia arjuna* mediated SeNPs gel holds promise as a novel and effective approach for addressing the challenges associated with chronic periodontitis treatment.

## Patients consent

As this is an in-vitro study patient's consent is not applicable.

## Author statement

Contributor 1: Concepts, Literature search, Data analysis, Statistical analysis, Manuscript preparation, Guarantor, Contributor 2: Design, Definition of intellectual content, Clinical studies, Experimental studies, Data acquisition, Manuscript editing, Manuscript review, Guarantor, Contributor 3: Concepts, Literature search, Data analysis, Manuscript editing, Guarantor, Contributor 4: Design, Definition of intellectual content, Clinical studies, Experimental studies, Data acquisition, Statistical analysis, Manuscript preparation, Manuscript review, Contributor 5: Concepts, Literature search, Data analysis, Manuscript editing, Contributor 6: Design, Literature search, Experimental studies, Statistical analysis, Manuscript review.

## Funding

There is no source of funding for this study.

## Declaration of competing interest

The authors declare that they have no known competing financial interests or personal relationships that could have appeared to influence the work reported in this paper.

## References

[1] Mohideen K., Chandrasekar K., Ramsridhar S., Rajkumar C., Ghosh S., Dhungel S. (2023 May 30). Assessment of oxidative stress by the estimation of lipid peroxidation marker malondialdehyde (MDA) in patients with chronic periodontitis: a systematic review and meta-analysis. Int J Dent.

[2] Tsimpiris A., Tsolianos I., Grigoriadis A., Moschos I., Goulis D.G., Kouklakis G. (2023 May). Association of chronic periodontitis with Helicobacter pylori infection in stomach or mouth: a systematic review and meta-analysis. Eur J Dermatol.

[3] Chatzopoulos G.S., Koidou V.P., Tsalikis L. (2023 Mar). Local drug delivery in the treatment of furcation defects in periodontitis: a systematic review. Clin Oral Invest.

[4] Viglianisi G., Santonocito S., Lupi S.M. (2023 Sep 13). Impact of local drug delivery and natural agents as new target strategies against periodontitis: new challenges for personalized therapeutic approach. Ther Adv Chronic Dis.

[5] Budală D.G., Luchian I., Tatarciuc M. (2023 Jun 19). Are local drug delivery systems a challenge in clinical Periodontology?. J Clin Med.

[6] Hatila S., Lahiri B., Sunnanguli G. (2023 Mar 1). Comparative assessment of the effect of three various local drug delivery medicaments in the management of chronic periodontitis. J Contemp Dent Pract.

[7] Tharani M., Rajeshkumar S., Al-Ghanim K.A., Nicoletti M., Sachivkina N., Govindarajan M. (2023). *Terminalia chebula*-assisted silver nanoparticles: biological potential, synthesis, characterization, and ecotoxicity. Biomedicines.

[8] Nasiri K., Masoumi S.M., Amini S. (2023). Recent advances in metal nanoparticles to treat periodontitis. J Nanobiotechnol.

[9] Kiarashi M., Mahamed P., Ghotbi N. (2024). Spotlight on therapeutic efficiency of green synthesis metals and their oxide nanoparticles in periodontitis. J Nanobiotechnol.

[10] Benedini L. (2023). Advanced and Modern Approaches for Drug Delivery.

[11] Clément S., Winum J.Y. (2024 Jun). Photodynamic therapy alone or in combination to counteract bacterial infections. Expert Opin Ther Pat.

[12] Devi K., Paulraj J., George R.S. (2024). A comparative in vitro analysis of antimicrobial effectiveness and compressive resilience in Chirata and Terminalia arjuna modified glass ionomer cement. Cureus.

[13] Reddy R.N., Dandu S.P., Sravanthi G., Mohammed S., Narahari S., Sistla S. (2020). Terminalia arjuna – a possible alternative to commercial mouthwashes, against periodontopathic bacteria: an in vitro study. J Dr NTR Univ Health Sci.

[14] Puri A., Mohite P., Patil S. (2023 Sep 22). Facile green synthesis and characterization of *Terminalia arjuna* bark phenolic-selenium nanogel: a biocompatible and green nano-biomaterial for multifaceted biological applications. Front Chem.

[15] Moghaddam F.D., Heidari G., Zare E.N. (2023 Mar 15). Carbohydrate polymer-based nanocomposites for breast cancer treatment. Carbohydr Polym.

[16] Arslaan M., Karim N., Kadri W.B., Asghar S. (2021). Local drug delivery to treat chronic periodontitis. J Bahria Univ Med Dent Coll.

[17] Kachhadiya P., Duseja S., Parikh H., Patel Z. (2022). Evaluation of local drug delivery system containing 1% (W/V) chitosan gel used as an adjunct to scaling and root planning in chronic periodontitis– A split mouth study. Int J Dent Res.

[18] Au A., Mojadadi A., Shao J.Y., Ahmad G., Witting P.K. (2023 Mar 23). Physiological benefits of novel selenium delivery via nanoparticles. Int J Mol Sci.

[19] Madi M., Pavlic V., Samy W., Alagl A. (2018 Jan). The anti-inflammatory effect of locally delivered nano-doxycycline gel in therapy of chronic periodontitis. Acta Odontol Scand.

[20] Ghosh P. (2023). A review on nano-approaches against periodontitis treatment. Global Journal of Pharma and Paramedical Research.

[21] Hamman N., Ramburrun P., Dube A. (2024 Mar 25). Selenium nanoparticle activity against *S. mutans* biofilms as a potential treatment alternative for periodontitis. Pharmaceutics.

[22] Ismikhanov A.G., Dadaeva G.T., Dzhabrailov S.M., Maysigova J.B., Semenov M.R., Dzagurova L.A. (2023). Prospects for the use of selenium-containing drugs in dentistry. Ann Dent Spec.

[23] Hou J., Tamura Y., Lu H.-Y. (2022). An in vitro evaluation of selenium nanoparticles on osteoblastic differentiation and antimicrobial properties against *Porphyromonas gingivalis*. Nanomaterials.

[24] Uskoković V., Pejčić A., Koliqi R., Anđelković Z. (2022). Polymeric nanotechnologies for the treatment of periodontitis: a chronological review. Int J Pharm.

[25] Mahale S.A., Dhadse P. (2023). Assessment and comparison of the antimicrobial and clinical efficacy of copper nanoparticles gel with scaling and root planing (SRP) against chlorhexidine gel with scaling and root planing (SRP) as a local drug delivery agent in patients with periodontitis –A randomized interventional clinical trial. Research Square.

[26] Mutthineni R.B., Himaja G., Paul R.A., Muralidharan G., Burli V.V.A., Duddukuri H. (2023). Evaluation of different local drug delivery systems in the management of chronic periodontitis: a comparative study. IP Int J Periodontol Implantol.

[27] Amato M., Santonocito S., Polizzi A. (2023 Apr 21). Local delivery and controlled release drugs systems: a new approach for the clinical treatment of periodontitis therapy. Pharmaceutics.

[28] Chen H., Zhang Y., Yu T. (2022 Oct 21). Nano-based drug delivery systems for periodontal tissue regeneration. Pharmaceutics.

[29] Mahadlek J., Charoenteeraboon J., Choopun S., Phaechamud T. (2010). Role of zinc oxide on rheology of Thermosensitive gel developed for periodontitis treatment. Adv Mater Res.

[30] Dabhi M.R., Nagori S.A., Gohel M.C., Parikh R.K., Sheth N.R. (2010). Formulation development of smart gel periodontal drug delivery system for local delivery of chemotherapeutic agents with application of experimental design. Drug Deliv.

[31] Vedernikova Iryna (2015). Synthesis and evaluation of zinc substituted magnetite nanoparticles for drug delivery systems. Int J Pharm Pharmaceut Sci.

